# Insights into the Mechanism of Enhanced Dissolution in Solid Crystalline Formulations

**DOI:** 10.3390/pharmaceutics16040510

**Published:** 2024-04-07

**Authors:** Anna Justen, Gerhard Schaldach, Markus Thommes

**Affiliations:** Laboratory of Solids Process Engineering, Department of Biochemical and Chemical Engineering, Technical University Dortmund, Emil-Figge-Straße 68, 44227 Dortmund, Germany; anna.justen@tu-dortmund.de (A.J.);

**Keywords:** dissolution mechanism, dissolution enhancement, formulation strategy, dissolution, flow through cell

## Abstract

Solid dispersions are a promising approach to enhance the dissolution of poorly water-soluble drugs. Solid crystalline formulations show a fast drug dissolution and a high thermodynamic stability. To understand the mechanisms leading to the faster dissolution of solid crystalline formulations, physical mixtures of the poorly soluble drugs celecoxib, naproxen and phenytoin were investigated in the flow through cell (apparatus 4). The effect of drug load, hydrodynamics in the flow through cell and particle size reduction in co-milled physical mixtures were studied. A carrier- and drug-enabled dissolution could be distinguished. Below a certain drug load, the limit of drug load, carrier-enabled dissolution occurred, and above this value, the drug defined the dissolution rate. For a carrier-enabled behavior, the dissolution kinetics can be divided into a first fast phase, a second slow phase and a transition phase in between. This study contributes to the understanding of the dissolution mechanism in solid crystalline formulations and is thereby valuable for the process and formulation development.

## 1. Introduction

In the formulation development of oral solid dosage forms, one challenge is the low aqueous solubility of many drugs [1,2]. Different approaches aim to enhance the dissolution rate in water, such as solubilization techniques, chemical modifications, particle size reduction or the formulation of solid dispersions [3,4,5]. The last is a promising approach for the improvement of drug dissolution, which has been highly investigated in the past [6,7]. Solid dispersions were first categorized according to Chiou and Riegelman [8]. Due to the rise of new formulation approaches for solid dispersion, the definition of solid dispersions was extended [6]. They have been described as dosage forms, which show an increased drug dissolution compared to the pure drug, caused by the insertion of that drug into a hydrophilic carrier [9].

The dissolution enhancing factors are intensively discussed and mechanisms explaining the increased drug dissolution rate are proposed [6,7,10,11]. The drug dissolution was found to be either carrier- or drug-controlled [9]. It is assumed that during dissolution, a high concentrated layer of dissolved carrier material is formed at the dissolving front. In carrier-controlled dissolution, the drug dissolves into this carrier-rich layer [9]. This is indicated by a high increase in drug solubility in solutions of the carrier compared to solubility in water [12,13]. Thus, the dissolution rate of the drug in the bulk solution solely depends on the dissolution rate of the carrier material. In drug-controlled dissolution, the drug does not dissolve into the carrier-rich layer, but instead is directly released into the bulk. Hence, the dissolution rate is defined by the drug and drug particle properties, such as particle size.

A particle size reduction or increase in the particle surface *A*, which is wetted by the solvent during dissolution, will result in an increased dissolution rate $\frac{dm}{dt}$, as it is described by the law of Nernst and Brunner [14,15]:

(1) $$\frac{dm}{dt}=\frac{D\cdot A}{\delta }(c_{s}-c_{t})$$

where *D* is the diffusion coefficient of the dissolving component in the solvent, *δ* is the diffusion layer thickness, *c_s_* is the saturation concentration of the dissolving compound in the solvent and *c_t_* is the concentration after time, *t*. This model is suitable to describe the dissolution of pure drug particles. For solid dispersions with a crystalline sugar alcohol serving as carrier and the poorly soluble drug griseofulvin (drug load w = 10 wt.%), a linear decrease in the mean dissolution time (MDT) was found for decreasing mean particle sizes of the drug [16].

In addition to the particle size, the dissolution mechanism of solid dispersions also depends on the drug load [17]. For amorphous solid dispersions, two mechanisms of drug load depending drug dissolution were found. Below a threshold drug load, the drug dissolution was fast, and the drug and polymer dissolved congruently. Above this so-called Limit of Congruency, the drug dissolution was slow and independent of the polymer [11]. The decrease in drug dissolution rate above the limit of congruency has been explained by the formation of a hydrophobic continuous phase of drug, which prevents its release into the bulk [10]. This was also observed for solid dispersions with mannitol as the carrier material, prepared via co-milling with an air jet mill [18]. In that work, the decrease in dissolution rate above a threshold drug load was explained with a coherent cluster of drug particles, which developed due to percolation. For the co-milled formulations, the percolation threshold was presumed to be above a drug load of 10 wt.%. In amorphous formulations with copovidone as the carrier material, a variation between the Limit of Congruency of 5 and 40 wt.%, depending on the drug, process and particle size was found [11].

Besides the properties of the solid dispersion itself, the hydrodynamic conditions defined by the dissolution apparatus and the volume flow are helpful means in understanding the dissolution mechanism. The flow through cell (apparatus 4) [19] is a suitable dissolution method for poorly soluble pure drugs, powder and granule formulations [19,20,21]. Reproducible dissolution profiles can be obtained due to a homogeneous flow field [21,22,23]. The flow velocity can be adjusted by the pump rate; it was found that a pulsating pump causes variations in the hydrodynamics and thus the dissolution kinetics [24,25]. Increasing dissolution rates were found with increasing flow rates in the flow through cell [26,27].

As discussed above, different approaches aimed to explain the fast dissolution of a drug from solid dispersions. The main share of research has focused on amorphous solid dispersions [7,11,28].

One challenge in these amorphous solid dispersions is the thermodynamic instability of the amorphous form and the high effort in compatibility testing of drugs and polymers [6]. Formulating a solid dispersion with a crystalline drug and carrier, these disadvantages can be overcome. In the past, solid crystalline suspension showed a fast dissolution of poorly soluble drugs, as well as long-term stability and compatibility of crystalline sugar alcohols with several drugs [18,29]. Different manufacturing methods were presented, such as hot melt extrusion [30,31,32], co-milling [18], melt electrostatic precipitation [33,34,35] and melt milling [16].

In these studies, the dissolution mechanism was investigated and an improved particle wetting due to the hydrophilic surrounding was found to be one main reason for the dissolution enhancement [18]. This was confirmed by contact angle measurements, where a higher surface free energy was calculated for the co-milled solid dispersion. Furthermore, Raman mapping was conducted to evaluate the particle dispersion, with a high particle dispersity in the carrier [18]. Next to binary solid crystalline suspensions, the influence of the addition of sodium dodecyl sulfate on particle agglomeration and drug dissolution was investigated. Foaming occurred during the milling process, which prevented particle agglomeration as drug particles were assumed to assemble at the interface of the gas bubbles and the xylitol [36].

To exclude effects of the manufacturing method and variations in the solid state of the drug and the carrier, physical mixtures of different drugs and xylitol were prepared in this study to investigate the dissolution mechanism. The dissolution rate constant of the Hixson–Crowell equation was utilized to evaluate the effect of xylitol on the dissolution kinetics. For this purpose, the drug load, hydrodynamics in the flow through cell, solubility of drug in a xylitol-rich environment and the particle size were investigated. Finally, a dissolution mechanism was proposed for carrier-enabled and drug-controlled dissolution.

## 2. Materials and Methods

The poorly water-soluble drugs celecoxib (HangZhou Yuhao Chemical Technology, Hangzhou, China), naproxen (Alfa Aesar, Haverhill, MA, USA) and phenytoin (Recordati Pharma, Ulm, Deutschland) were investigated as physical mixtures. Due to their categorization as BCS class II drugs, bioavailability can be improved by dissolution-enhancing formulation techniques [37]. Furthermore, these drug substances are known for their highly stable crystalline form and low tendency to amorphize [38,39,40].

**Figure 1:**
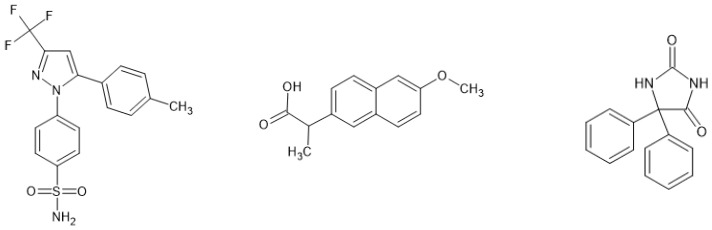
Chemical structures of celecoxib (**left**), naproxen (**middle**) and phenytoin (**right**).

The chemical structures are shown in Figure 1.

Xylitol (Roquette, Lestrem, France) was mixed with different drug contents (0.5, 1 and 5 wt.%) in a turbula mixer (Turbula T10B Mixer, Willy A. Bachofen, Muttenz, Switzerland).

To investigate the effect of particle size reduction, the drug and xylitol particles were co-milled in a planetary ball mill (TYP S-1, Retsch GmbH, Haan, Germany). A grinding cup was 1/3 filled with 20 mm corundum grinding balls and 1/3 with a physical mixture containing 1 wt.% drug. A rotational intensity of 50% was adjusted. The grinding process was stopped every 2 min and left to cool down for 10 min to prevent a heating of the grinding material and thereby degradation or phase transition. The grinding process was conducted for 2, 5, 10, 25 or 30 min and samples of each grinding stage were collected. For each sample, the particle size distribution was determined, differential scanning calorimetry (DSC) measurements were conducted and the in vitro dissolution was tested.

The particle size distribution was determined via laser diffraction measurement (Mastersizer 3000, Malvern, UK) and evaluated with the Mie theory. The Hydro SV device was used with a stirrer speed of 800 U min^−1^.

DSC measurements were conducted to confirm the crystallinity of the compounds of the physical mixtures and the co-milled compounds. 6–10 mg of the pure drug, pure xylitol and the co-milled physical mixture were prepared in an aluminum pan. The heating rate was set to 10 K/min. The melting points can be found in Table 1. Celecoxib was found in the stable form III [41], naproxen in its stable form I [42] and phenytoin and xylitol in the stable crystal form [43,44].

The in vitro dissolution experiments were conducted according to the method described in the *Pharmacopeia Europaea* with the flow through cell (22.6 mm tablet cell, apparatus 4, Erweka, Langen, Germany) in a closed loop [19]. The samples were placed on top of 1 mm glass beads. Purified water was heated to 37 °C and pumped with a gear pump (Ismatec 183, Wertheim, Germany) to avoid disturbances by pump pulsation. The absorption was measured continuously with a 50 mm cuvette in a UV-Vis spectrometer (Lambda 2s, Perkin Elmer, Überlingen, Germany).

Next to the particle size, the influence of drug load of the physical mixtures on the dissolution behavior was investigated. Drug contents of 0.5, 1 and 5 wt.% were tested as well as the pure drug and the pure xylitol. To keep the initial dissolving surface constant for each measurement, mini tablets with a diameter of 3 mm and a weight of 20 mg were prepared (Type 3, Paul Weber, Bösingen, Germany). Compression force of 7 kN was kept for 30 s. As xylitol has a weak UV traceability, the dissolution experiments were conducted as open-loop setup with a more sensitive UV–Vis spectrometer (Specord 200 Plus, Analytik Jena AG, Jena, Germany). To evaluate the influence of hydrodynamics in the flow through cell, pump rates between 4 mL/min and 42 mL/min were set.

The solubility of each drug in aqueous xylitol solutions of different concentrations was determined. Xylitol solutions with concentrations of 0.26 kg/L, 0.45 kg/L and the saturation concentration (0.64 kg/L) [45] were prepared and an excess of drug powder was added. The suspensions were tempered at 37 °C for 72 h, where they were frequently agitated to redisperse the floated drug crystals. Afterwards, the suspensions were filtrated with a syringe filter (Polypropylene, 0.45 µm, VWR, Radnor, PA, USA) and diluted for the following UV measurement (Lambda 2s, Perkin Elmer, Überlingen, Germany).

The Hixson–Crowell model was applied by calculating the Hixson–Crowell constant *K_HC_* for each dissolution curve [46].

## 3. Results and Discussion

### 3.1. Influence of Drug Load on Drug Dissolution

**Figure 2:**
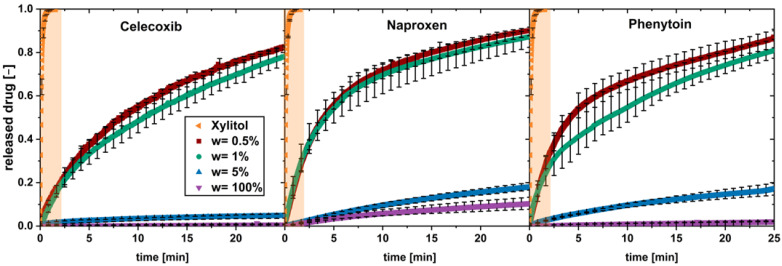
In vitro dissolution profiles of physical mixtures with different drug loads (w) and pure xylitol (orange plane) (av ± min/max; *n* = 3).

In solid formulations with a poorly water-soluble API and a highly soluble carrier material, the drug load was found to be a crucial parameter for the drug dissolution [9,17]. To investigate this for low drug loads up to 5 wt.%, physical mixtures, containing poorly soluble drugs with xylitol as carrier, were tested in the flow through cell. The drug particle size and volume flow ($\dot{V}=28\frac{mL}{min}$) were kept constant (Figure 2).

A fast dissolution was observed for low drug loads of 0.5 and 1 wt.%, while for the higher drug load of 5 wt.% and the pure drug, a slow dissolution was found.

The dissolution profiles for the fast-dissolving physical mixtures (w = 0.5 wt.% and w = 1 wt.%) can be divided into two phases showing a constant dissolution rate. The first phase shows a fast dissolution in the first minute; a second phase occurred above 5 min, with slower dissolution rate. A transition phase can be seen between both phases.

The dissolution profile of pure xylitol is added to each diagram and highlighted in orange. Xylitol dissolves fast and completely during the first 1.5 min.

Regarding dissolution of the 5 wt.% physical mixtures and the pure drugs, a single phase of constant, slow drug dissolution can be observed. These observations apply for all the three tested drugs.

In conclusion, the limit of drug load lies between a drug load of 1 and 5 wt.%. It can be assumed that, above this limit of drug load, the carrier xylitol dissolves fast, and a coherent cluster of hydrophobic drug is built when water penetrates into the system.

Below this limit of drug load, a fast drug dissolution occurred. The dissolution rate of the drug was not found to be equal to the fast-dissolving xylitol, hence the dissolution could not be described as being congruent as described in the literature [9,11]. Nevertheless, the presence of xylitol in the environment of dissolving drug particles is supposed to enable a fast dissolution. This behavior is called “carrier-enabled” subsequently.

Further investigations were conducted, considering the hydrodynamics during dissolution.

### 3.2. Investigation of the Dissoltuion Kinetics via Hydrodynamics

The dissolution mechanism was further analyzed by altering the hydrodynamic conditions during dissolution. Therefore, the volume flow was varied in the flow through cell, and the Reynolds number, *Re*, was calculated for each volume flow according to

(2) $$Re=\frac{\rho \cdot v\cdot L}{\eta }$$

It depends on the liquid density ρ, the fluid velocity v, the dynamic viscosity η and the characteristic length, which is the diameter of the flow through cell. The Reynolds number is a dimensionless number, which is used to describe the flow regime in the flow through cell. Due to the low investigated Reynolds numbers, a laminar flow regime can be assumed, where a homogenous flow profile is obtained in the flow through cell (diameter of 22.6 mm) [47].

**Figure 3:**
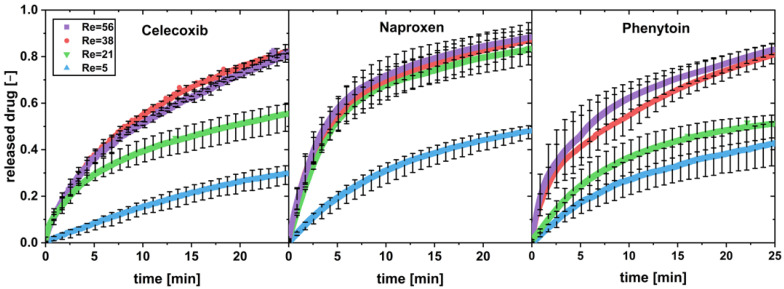
In vitro dissolution profiles of physical mixtures with a drug load of w = 0.5 wt.% with different Reynolds numbers (Re) in the flow through cell (apparatus 4) (av ± min/max; *n* = 3).

The dissolution profiles, exemplarily for a drug load of 0.5 wt.%, are shown in Figure 3.

The increase in the Reynolds number shows a dissolution-rate-increasing effect for all three poorly soluble drugs below a drug load of 5 wt.% (exemplarily shown in Figure 2 for 0.5 wt%). Variations in the tablet disintegration time due to higher flow rates in the flow through cell may lead to faster dissolution as seen in naproxen (Re = 21).

Furthermore, the Hixson–Crowell cube root law was considered [46]:

(3) $$m_{0}^{\frac{1}{3}}-m_{R}^{\frac{1}{3}}=K_{HC}\cdot t$$

The model describes the dissolution of one particle, with respect to the surface area decrease after time *t*. The initial particle mass is *m_0_*, the remaining particle mass of the dissolving particle is *m_R_* and *K_HC_* is the Hixson–Crowell dissolution rate constant.

**Figure 4:**
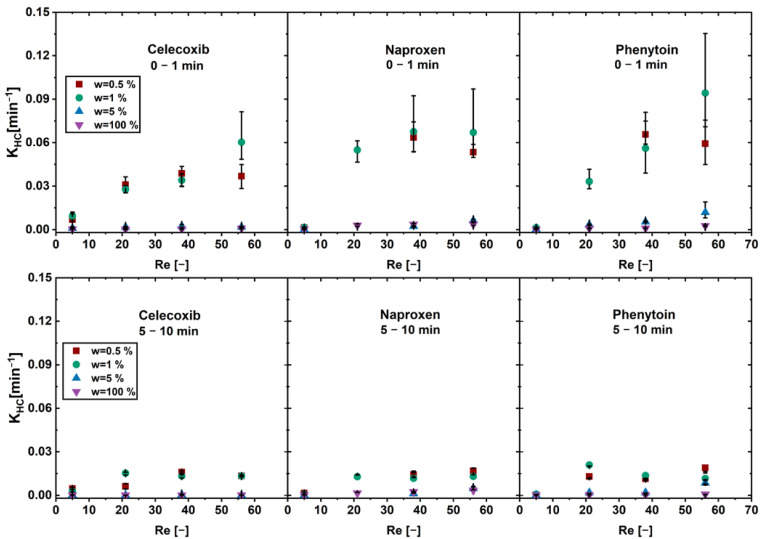
*K_HC_* of the first dissolution phase (0–1 min) and the second phase (5–10 min) as a function of the Reynolds number (*Re*) in the flow through cell for physical mixtures of different drug loads w (av ± min/max; n = 3).

The model is derived from the Nernst and Brunner equation and is suitable for the prediction of the dissolution of a pure API particle. Hence, *K_HC_* is constant when pure drug dissolves or a drug-controlled dissolution is observed. In the case of carrier-enabled dissolution, where a fast first phase and a slow second phase occurs, *K_HC_* alters throughout the dissolution process. A constant, high value for the first phase, a low value for the second phase, and altering values for the transition region are obtained. *K_HC_* was determined for the first and the second phase of the physical mixtures and is depicted as a function of the Reynolds number (Figure 4). The first phase was defined as the first minute of dissolution and the second phase was defined as minute 5–10.

An increase in the *K_HC_* over the Reynolds number was found for the physical mixtures below the limit of drug load for all drugs in the first phase. Above the limit of drug load and for the pure drug, an increase in convection (Reynolds number) did not lead to an increase in dissolution rate.

In the second phase no increase in the *K_HC_* was found for all drug loads. The physical mixtures below the limit of drug load showed overall higher *K_HC_* than the pure drug and the physical mixtures above the limit of drug load.

The application of increased convection emphasizes the presence of two distinct dissolution mechanisms for physical mixtures below and above the limit of drug load. Since the xylitol content plays an important role in dissolution enhancement, its influence on the dissolution kinetics was further investigated and described in the following.

### 3.3. Influence of Xylitol on the Dissolution Kinetics

The carrier- and drug-controlled mechanisms, which are described in the literature, are mainly investigated for amorphous formulations [9] and are discussed for the present crystalline systems. Focusing on the carrier-controlled dissolution mechanism, a congruent dissolution of the drug and the carrier were observed [9,11]. However, a clear categorization cannot be carried out for the present system. This is based on the typical criteria described in the literature: xylitol dissolves much faster than any tested drug, the different drugs do not show the same dissolution rate in the presence of xylitol and the dissolution rate is drug-load-dependent. Consequently, a congruent dissolution of the drug and carrier cannot describe the present xylitol-containing formulations.

Besides this, a carrier-controlled dissolution behavior as described in the literature is characterized by a high solubility of the drug in concentrated carrier solutions [9]. To validate this for the present system, the solubility of the drugs was measured in different xylitol solutions. The measurements were conducted in triplicate. The determined values are listed in Table 2.

No significant (α > 0.05) increase in drug solubility with increasing xylitol concentration in the drug environment could be observed based on a regression analysis.

Compared to the findings in the literature, a high linear increase would be expected as it was seen for another crystalline carrier [13]. A linear or sigmoidal increase in drug solubility was also found for formulations with polyvinylpyrrolidone as carrier material with a factor of solubility enhancement of up to 30 [12,48].

It can be concluded that the increase in the dissolution rate for low drug loads cannot be attributed to a solubility-enhancing environment during dissolution.

Above the limit of drug load, a drug-controlled dissolution behavior was identified, which is explained with the formation of hydrophobic drug clusters, so that the dissolution-rate-enhancing influence of xylitol vanished.

Although no congruent behavior was identified, xylitol enables a fast drug dissolution in the first phase and second phase. The transition area in between was not evaluated, as no fixed rate constant (*K_HC_*) could be determined. To investigate the dissolution kinetic over the entire period of dissolution, the mean dissolution time (MDT) was calculated for each dissolution curve [49]. It only applies for completed dissolution and was therefore calculated for the physical mixtures below the limit of drug load:

(4) $$MDT=\frac{\sum_{i=1}^{n}t_{i}\cdot ∆m_{i}}{\sum_{i=1}^{n}∆m_{i}}$$

If the dissolution of the drug is carrier-enabled, an increase in convection will increase the dissolution of the carrier xylitol and the drug in a physical mixture in the same extent, so that the ratio remains constant for the different Reynolds numbers.

**Figure 5:**
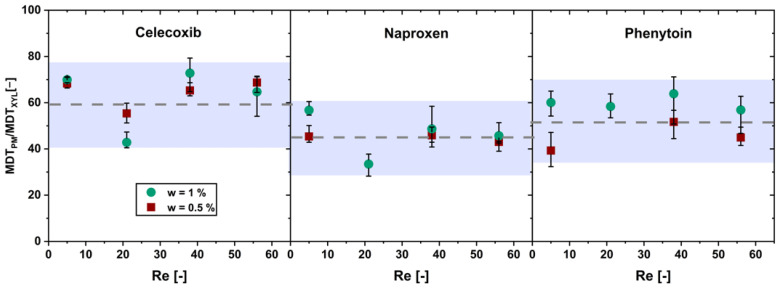
Ratio of the mean dissolution time (MDT_PM_) of the physical mixture with drug content w and the MDT of pure xylitol (MDT_XYL_) over the Reynolds number (*Re*) in the flow through cell (av ± min/max; *n* = 3).

Figure 5 shows the ratio of the MDTs of the physical mixtures below the limit of drug load and the pure xylitol as a function of the Reynolds number.

The ratio shows no significant increase (α > 0.05) in the MDT ratio over the Reynolds number for the physical mixtures below the limit of drug load. Hence, a xylitol-dependent dissolution of the physical mixtures below the limit of drug load was confirmed.

In conclusion, the dissolution behavior of fast-dissolving formulations with a high xylitol content (w < limit of drug load) can be distinguished in a first fast phase, a second slower phase and a transition region in between. The drug dissolution was found to be enabled by xylitol over the entire dissolution period. Therefore, the influence of drug particle size in xylitol containing physical mixtures was investigated in the following.

### 3.4. Particle Size

**Figure 6:**
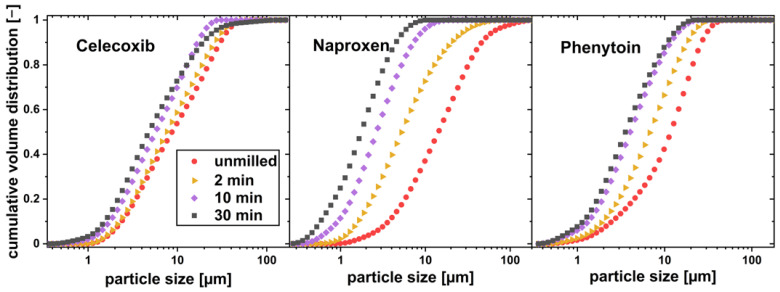
Particle size distribution of drug particles co-milled with xylitol in a planetary ball mill for different milling times.

To investigate the influence of particle size reduction on the dissolution behavior of physical mixtures below the limit of drug load, xylitol, and the drugs celecoxib, naproxen and phenytoin were co-milled. The particle size distribution of the drug particles measured after different milling times can be found in Figure 6.

**Figure 7:**
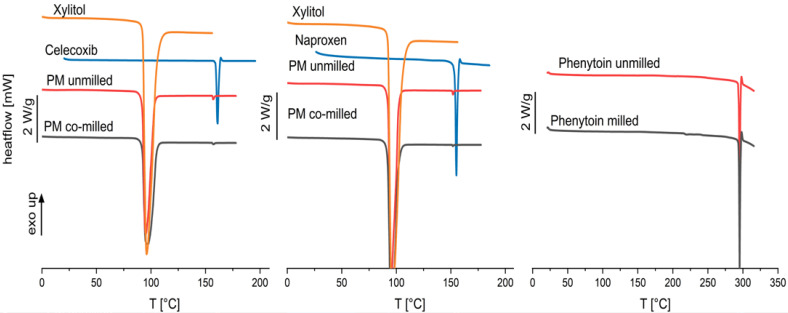
Thermograms of pure xylitol, pure drug, physical mixtures of celecoxib and naproxen unmilled and co-milled, and phenytoin unmilled and milled.

The particle sizes of all drugs could be reduced by co-milling, where the milling process was limited by particle agglomeration in the milling cup. Celecoxib and phenytoin physical mixtures agglomerated after more than 20 min of milling, where naproxen containing physical mixtures showed no agglomeration even after 30 min of milling. No phase change or instable crystal form was found by the DSC measurement, as no glass transition was found for any of the drugs. The thermograms can be seen in Figure 7. As phenytoin has a particularly high melting temperature, exceeding the melting temperature of xylitol, a physical mixture cannot be investigated via DSC. Therefore, phenytoin was milled for 30 min without xylitol and DSC measurements were conducted.

The thermograms indicate crystallinity of the physical mixtures after co-milling (celecoxib and naproxen) and single milling (phenytoin) due to there being no occurrence of a glass transition as well as a melting peak. A negligible melting point depression is observed for celecoxib and naproxen physical mixtures which indicates a low solubility of the API in the carrier xylitol. However, the chosen model drugs have been categorized in glass-forming ability class I and II, characterized by a low-amorphization tendency [38,39,40].

**Figure 8:**
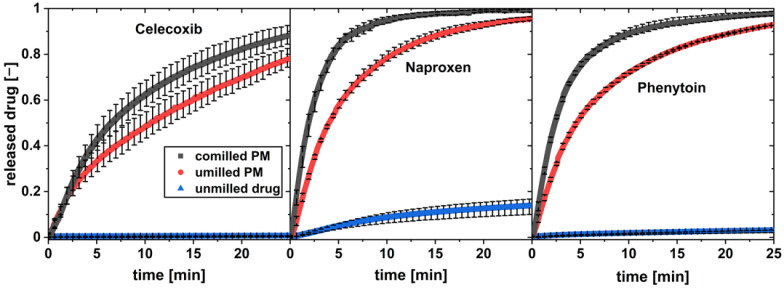
In vitro dissolution profiles of co-milled physical mixture, unmilled physical mixture (PM), and pure drug at a flow rate of 16 mL/min and a drug load of w = 1 wt.% (av ± min/max; *n* = 3).

To evaluate the influence of the particle size reduction on the dissolution behavior of the pure drug, the unmilled physical mixture and the co-milled physical mixture were tested in the flow through cell. The volume flow in the flow through cell ($\dot{V}=16\frac{mL}{min},\;Re=21$) and the drug content was set constant for each measurement (w = 1 wt.%). The dissolution profiles can be seen in Figure 8.

All three unmilled drugs show practically low dissolution in water over the observed time. For all drugs, a high increase in the drug dissolution rate was observed for the physical mixture of drug and xylitol. The co-milled physical mixture with the highest milling time and thereby highest drug surface area S_A_ was tested for each drug. It showed a further increase in the drug dissolution compared to the unmilled physical mixture.

**Figure 9:**
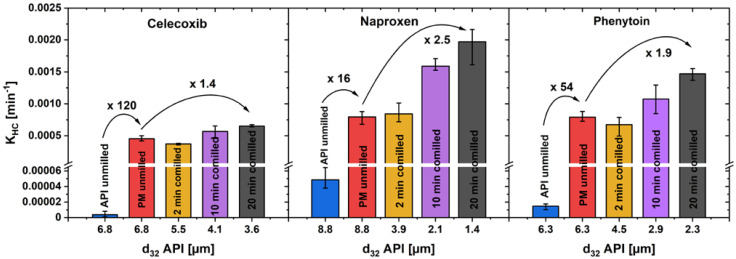
*K_HC_* in the first phase of drug dissolution, over the Sauter mean diameter of the drug particles of pure drug powder, unmilled and co-milled physical mixtures (av ± min/max; *n* = 3).

A comparison of the *K_HC_* for unmilled drug, unmilled physical mixture and three milling steps of co-milled physical mixture in the first dissolution phase can be found in Figure 9. As a representative particle size can be calculated with the Sauter diameter *d*_32_, which is defined as

(5) $$d_{32}=\frac{6}{S_{A}}$$

For unmilled drug and physical mixture, the Sauter diameter of the drug is equal, as the unmilled physical mixture was prepared by simple powder-mixing without comminution. The *K_HC_* of unmilled drug, compared to unmilled physical mixture, increased with a remarkably high factor of 120 for celecoxib, 16 for naproxen and 54 for phenytoin, while co-milling only further enhanced *K_HC_* by a factor of up to 2.5. An increased drug particle wetting by the addition of a hydrophilic carrier and its fast dissolution in the direct particle environment is assumed to be one explanation for this observation [18]. A particularly high increase in the dissolution rate can be achieved by the addition of xylitol, so that the extra particle-processing step of co-milling can be omitted.

### 3.5. Dissolution Mechanism

**Figure 10:**
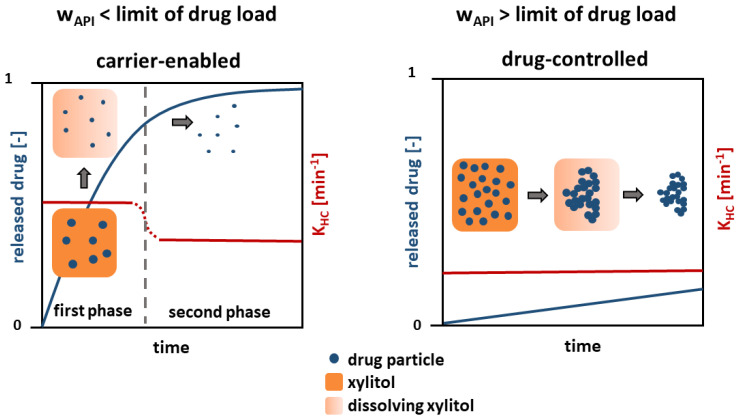
Schematic of the drug dissolution from solid crystalline formulations.

A mechanism is proposed based on the findings, which describes the dissolution of the presented poorly water-soluble model drug substances from a solid crystalline formulation (Figure 10).

Regarding the dissolution behavior below the limit of drug load (≤5 wt.%), a carrier-enabled drug dissolution is proposed. A fast drug dissolution rate was found in the first phase, which is caused by the simultaneous, but not congruent, dissolution of xylitol and drug. A high content of xylitol in the formulation facilitates wetting of each single drug particle [18], so that a fast drug dissolution occurs. Particle agglomeration is prevented due to the low particle concentration and fast wetting of the entire particle surface.

In the second phase, after the complete dissolution of the xylitol, a low dissolution rate constant was found. Small, separated drug particles result, whose dissolution solely depends on the drug and the particle size. However, a dissolution rate constant slightly faster than that for pure drug and physical mixtures above the limit of drug load (≥5 wt.%) was observed in the second phase. It is assumed that this is due to particle size effects. In the first phase, drug particle dissolution begins, where the particle size is reduced and the specific surface area increases, leading to a higher dissolution rate constant in the second phase.

Above the identified limit of drug load (≥5 wt.%), the mechanism changes and the dissolution is drug-controlled. A slow dissolution rate, comparable to that of pure drug was found. A complete wetting of the separated particles enabled by xylitol is prevented due to the high drug-particle concentration. A cluster of hydrophobic drug particles develops, which has a low, outer specific surface area and thus dissolves slowly.

A limit of drug load and a mechanism describing the dissolution of drug from solid crystalline formulations comprising xylitol and a poorly water-soluble drug was not proposed before. The understanding of the dissolution mechanism facilitates the development of new formulations.

## 4. Conclusions

Solid dispersions are important formulation techniques to enhance the drug dissolution rate of poorly water-soluble drugs. Formulations with a crystalline carrier and drug are one approach, which show a fast dissolution and thermodynamic stability. In this study, the dissolution mechanisms leading to a faster dissolution of three model drug substances of BCS class II were investigated. Therefore, physical mixtures with the crystalline carrier xylitol were prepared. Different parameters were studied such as particle size and drug load, where the hydrodynamics were used as means to investigate the dissolution kinetics.

A limit of drug load between 1 and 5 wt.% was found, below which a fast, carrier-enabled dissolution occurred. It was found that three phases of dissolution could be distinguished, according to the dissolution rate constant *K_HC_*: a first fast phase, a transition phase and a second slower phase. It is assumed that the drug dissolution in the first phase is accelerated due to enhanced particle wetting by xylitol and thereby prevention of drug particle agglomeration. The second phase is defined by the dissolution of pure drug particles.

Above the limit of drug load the dissolution rate constant was low, and only one phase of dissolution was identified. The dissolution was found to be similar to that of pure drug and was described as drug-controlled. A high particle concentration in the formulation led to the development of hydrophobic clusters of drug lowering the outer specific surface area and thereby the dissolution rate constant.

In conclusion, this study provides a deeper understanding of the dissolution enhancing mechanisms in crystalline formulations containing three poorly water-soluble model drugs and xylitol as carrier material. It is likely that this also applies to other BCS class II drugs. However, molecular interactions during dissolution among drug, carrier and solvent cannot be excluded as a further factor in dissolution enhancement. Future research might aim on a deeper understanding of the dissolution mechanism on the molecular level. The knowledge of a limit of drug load, beyond which the dissolution mechanism changes, helps predict the bioavailability and lower the risk of dosing issues in new formulations.

## Figures and Tables

**Table 1:** Onset of melting points of the used substances (av ± SD; *n* = 3).

Substance	Melting Point [°C]
Celecoxib	159.8 ± 0.29
Naproxen	154.1 ± 0.25
Phenytoin	294.5 ± 0.11
Xylitol	92.5 ± 0.2

**Table 2:** Determined drug solubility in water and aqueous xylitol solutions (av (max/min); n = 3).

	Drug Solubility [mg/L]			
	Water	2.6 × 10^5^ mg/L Xylitol	4.5 × 10^5^ mg/L Xylitol	6.4 × 10^5^ mg/L Xylitol
Celecoxib	3.05 (0.21/0.41)	2.87 (0.01/0.04)	2.72 (0.03/0.02)	4.23 (0.47/0.29)
Naproxen	49.73 (2.30/1.62)	42.64 (1.76/158)	53.41 (0.84/1.49)	45.33 (1.60/1.36)
Phenytoin	37.74 (2.34/3.78)	37.58 (1.19/1.84)	33.85 (8.91/8.45)	36.82 (0.97/1.68)

## Data Availability

The data is available upon request by the corresponding author.
